# Intra-articular Injection Administration in UK Ex-professional Footballers During Their Playing Careers and the Association with Post-career Knee Osteoarthritis

**DOI:** 10.1007/s40279-019-01255-x

**Published:** 2020-01-10

**Authors:** Gwen S. Fernandes, Sanjay M. Parekh, Jonathan P. Moses, Colin W. Fuller, Brigitte E. Scammell, Mark E. Batt, Weiya Zhang, Michael Doherty

**Affiliations:** 1 Academic Rheumatology, Division of Rheumatology, Orthopaedics and Dermatology, School of Medicine, University of Nottingham, Clinical Sciences Building, Nottingham City Hospital, Nottingham, NG5 1PB UK; 2 Arthritis Research UK Centre for Sport, Exercise and Osteoarthritis, Queens Medical Centre, Nottingham, NG7 2UH UK; 3 Arthritis Research UK Pain Centre, Nottingham City Hospital, Nottingham, NG5 1PB UK; 4 Nottingham University Hospitals NHS Trust, Queen’s Medical Centre, Nottingham, NG7 2UH UK; 5 Colin Fuller Consultancy Ltd, Sutton Bonington, LE12 5PE UK

## Abstract

**Background:**

The long-term risk from knee intra-articular (KIA) injections in professional athletes such as ex-footballers remains unknown. The use of KIA injections is controversial and remains anecdotally prolific as it is perceived as being safe/beneficial. The aim of this study was to determine the number, type and frequency KIA injections administered to retired professional footballers during their playing careers and the associations with post-career knee osteoarthritis (KOA).

**Methods:**

This is a cross-sectional study involving a postal questionnaire (*n* = 1207) and subsequent knee radiographs in a random sample of questionnaire responders (*n* = 470). Footballers self-reported in the questionnaire whether they had received KIA injections and the estimated total number over the course of their playing career. Participant characteristics and football career-related details were also recorded. KOA was measured as self-reported knee pain (KP), total knee replacement (TKR) and radiographic KOA (RKOA).

**Results:**

44.5% of footballers had received at least one KIA injection (mean: 7.5; SD ± 11.2) during their professional career. 71% of knee injections were cortisone/corticosteroid based. Multivariate logistic regression, adjusting for age, body mass index (BMI) and significant knee injury identified that footballers with injections were two times more likely to have KP (OR 1.81, 95% CI 1.40–2.34) and TKR (OR 2.21, 95% CI 1.43–3.42) than those without injections. However, there was no association with RKOA (OR 1.30, 95% CI 0.85–2.01). Given, the association with KP and TKR, we found a significant dose–response relationship as the more injections a player received (by dose–response groups), the greater the risk of KP and TKR outcomes after adjustment for knee injury and other confounders (*p* for trend < 0.01).

**Conclusion:**

On average, 8 KIA injections were given to the ex-footballers during their professional career. The most commonly administered injections were cortisone based. These injections associated with KP and TKR after they retired. The associations are independent of knee injuries and are dose dependent. The study suggests that there may have been excessive use of KIA injections to expedite return to play and this contributed to detrimental long-term outcomes such as KP and TKR post-retirement from professional football.

## Key Points

**Table:** 

In this cross-sectional study of 1207 ex-professional footballers, over 44% reported receiving symptom-relieving knee intra-articular injections (predominantly cortisone based) during their professional careers. Even after adjustment for age, BMI and significant knee injury, there was a positive association between intra-articular injections and knee pain and total joint replacements, including a dose–response relationship with increasing number of injections.
This is the largest study of knee intra-articular injections received by ex-professional footballers and the findings suggest potential historic misuse of these injections in the English Football League and their detrimental impact on long-term knee osteoarthritis outcomes.

## Background

The use of intra-articular (IA) injection of local anaesthetic or corticosteroid to manage injury-related pain and enable early return to play has been reported in elite sport such as rugby league and Australian Rules Football [1], despite a lack of safety and efficacy data [2, 3]. The International Federation of Sports Medicine (FIMS) requires that a physician does not administer any treatment which may *‘*in any way mask pain to enable the athlete’s return to practising the sport if there is any risk of aggravating the injury’ [4]. Despite this recommendation, the risk from IA injections remains largely unknown and its use may be amplified due to the risk being perceived as less than the potential benefits [5, 6]. Gultekin et al. [7] recently reviewed the use of pain-relieving injections in sport and concluded there was some evidence of long-term safety issues. However, there were neither quantitative data nor robust evidence to support their conclusion and no specific data for knee joint injections in footballers.

In clinical practice, it is widely accepted that no more than four IA corticosteroid injections should be administered into the same joint in a 12-month period due to potential detrimental effects of excessive steroid on cartilage and other joint tissue [8]. In a randomised control trial, McAlindon and colleagues [9] found that regular use of IA corticosteroid injections was associated with greater cartilage volume loss but no significant difference in KP compared to IA placebo injections at 24-month follow-up. Anti-anabolic effects on healthy cartilage have also been reported particularly at high doses and durations of IA corticosteroid use [10]. The World Anti-Doping Agency (WADA) explicitly bans the use of any glucocorticoid such as cortisone whilst in competition and bans hyaluronic acid both in and out of competition [11]. However, in professional football, as with other elite sport, regulation of the administration of pain-relieving IA injection is limited and the type of injection, extent to which they are prescribed, and long-term follow-up have not been studied in detail [1, 6, 7]. Furthermore, WADA and their anti-doping regulations simply did not exist for elite footballers who played professionally during the 1950s–1980s. Data on use of knee IA (KIA) injections in professional footballers are limited to small studies (*n* = 27–100) in which 44–54% received knee IA (KIA) injections during their career and reported long-term impaired knee-health related quality of life, including 97% experiencing KP [12, 13].

Our previous study found that ex-professional footballers have more than a twofold increased risk of knee osteoarthritis (KOA) outcomes, specifically knee pain (KP), radiographic KOA (RKOA), and total knee replacement (TKR) compared to the general population [14]. The major risk factor associated with these KOA outcomes is knee injuries [15]. This study aimed to assess the number, type and frequency with which KIA injections have historically been administered to professional footballers during their careers in the UK, and to determine whether KIA injections are associated with KP, KOA and TKR, and whether the associations are independent of knee injuries.

## Methods

### Study Design

This is a cross-sectional study involving a postal questionnaire survey and subsequent radiographic assessment in a sample of responders [14]. The study was approved by the Nottingham Research Ethics Committee 1 (Refs 14/EM/0045 and 14/EM/0015). All procedures involving research participants were in accordance with the ethical standards of the Nottingham Research Ethics Committee and the University of Nottingham and with the 1964 Helsinki Declaration and its later amendments. Informed consent was obtained from all individual participants in this study.

### Participants

The inclusion criteria for the study were retired male professional footballers aged 40 years and older who held a professional contract within the top four tiers of the English Football League. Questionnaires were sent to footballers via a variety of football organisations and completed questionnaires returned to the Arthritis Research UK Centre for Sport, Exercise and Osteoarthritis, University of Nottingham. 4775 questionnaires were distributed, and 1207 questionnaires were completed and returned (25% response rate) [14]. Information was obtained on participant characteristics, details of professional football career and other known risk factors for KOA, including significant knee injury. Those footballers who indicated willingness to have knee radiographs, and who had not previously undergone bilateral TKR, were invited to attend their nearest Spire Healthcare hospital for knee radiographs. Out of the 860 individuals who provided consent for bilateral knee radiographs, 470 attended their local SPIRE HOSPITAL for a radiographic assessment (55% response rate) [14].

### Exposures

Receipt of KIA injections was assessed by the question ‘have you ever had any injections into your knees?’ If players replied positively, they were then asked to identify which knee was injected and the type of injection used with the following options: cortisone (steroid); anaesthetic (lignocaine); do not know or other (please specify). We also asked about the maximum number of KIA injections received in any season and the total number of injections into each knee over the course of their professional football career.

### Outcomes

KOA was measured via three outcomes; namely:

1. current KP, defined as *“*any pain for most days in the previous month”, was self-reported from the questionnaire, indicated on a body mannequin,
2. TKR was also self-reported in the questionnaire,
3. RKOA was determined in those respondents who were willing to undergo knee radiographs, which were undertaken as standardised bilateral weight-bearing semi-flexed (tibio-femoral compartments) and 30 degree flexed skyline (patello-femoral compartment) views, scored by a single assessor (GSF) using the Nottingham Line Drawing Atlas (NLDA) and Kellgren–Lawrence Scale. These methods, thresholds and associated reliability measures have been reported previously [14].

### Covariates

Age and body mass index (BMI) were self-reported in the questionnaire. Constitutional knee alignment (in early 20s) was self-reported and assessed using validated line-drawings [16]. In using this instrument, participants separately self-reported early adult life (early 20s—presumed to be constitutional) knee alignment as severe varus, mild varus, straight legs, mild valgus or severe valgus. Those with severe or mild varus were categorised as having a varus alignment, those with severe or mild valgus as having a valgus knee alignment and those with straight legs as neutral alignment. Footedness was assessed by asking players which foot they would use predominantly to kick a ball. Medication use and comorbidities such as gout were self-reported in the questionnaires. Career duration was derived as the time between the start of a professional football career (signed with a professional football club) and date of retirement from playing professional football (end of contract or retirement). Significant knee injury was defined as *“*one which caused pain for most days for at least a 3-month period and resulted in an absence from all training and matches during this time”. We treated significant knee injury as a *binary* variable in our subsequent analysis.

### Analysis

Descriptive analyses included *t* test for continuous variables and Chi-squared test for dichotomous/categorical variables. Associations between IA injections and KOA outcomes were determined using logistic regression and reported as odds ratio (OR) with a 95% confidence interval (CI) and adjusted for age, BMI and significant knee injury. A dose–response relationship was examined for each outcome in terms of groups of injections. We categorised total number of IA injections received into groups (0 injection = Group 1, 1–3 injections = Group 2, 4–6 injections = Group 3, 7 + injections = Group 4). We present the OR with 95% CI from logistic regression models using injection category (Groups 2–4) as our exposure variable in adjusted and unadjusted analyses with the referent group being Group 1, i.e., no KIA injections. Finally, we used the Stata command *nptrend* to perform a nonparametric test for trend across the injection groups for our three key outcomes: KP, TKR and RKOA [17]. We had little to no missing data and therefore, imputation methods were not used in these analyses. Data management and analysis were performed using Stata version 15.1 (StataCorp, College Station, TX, USA). Significance was determined at either the *p* < 0.05(*) or *p* < 0.01(**) level in the results.

## Results

Almost half (537, 44.5%) of the 1207 footballers who responded to the questionnaire had received IA injections into their knees over the course of their career (Table 1). The mean number of injections received was 7.5 (SD: 11.2) with a range from 1 to 100 for any one knee over a professional career. The mean number of injections into the right and left knees was 5.5 (SD: 7.2) and 5.6 (SD: 8.7), respectively. Of the 1207 footballers, 470 received bilateral knee radiographs that were used to determine NLDA and Kellgren Lawrence scores. RKOA in any knee using the NLDA scoring was present in over half (301, 64%) of ex-footballers.

**Table 1:** Ex-footballer characteristics including known risk factors for KOA outcomes

Characteristic	Received Injection		Total
	Yes (*n* = 537)	No (*n* = 670)	
Age (years), mean (SD)	58.6 (11.0)	59.3 (12.2)	59.0 (11.7)
BMI (kg/m^2^), mean (SD)**	27.5 (3.1)	27.0 (2.9)	27.2 (3.0)
2D:4D Ratio, *n* (%)	333 (64.9)	400 (62.6)	733 (63.6)
Malalignment^a^, *n* (%)	99 (18.6)	94 (14.5)	193 (16.4)
Right-footed, *n* (%)	314 (58.7)	399 (59.8)	713 (59.3)
Pain medication^b^, *n* (%)**	355 (66.1)	392 (58.6)	747 (61.9)
Gout, *n* (%)**	85 (15.8)	57 (8.5)	142 (11.8)
Significant injury^c^, *n* (%)**	440 (81.9)	338 (50.5)	778 (64.5)
Career duration (years), mean (SD)*	14.4 (5.2)	13.5 (5.8)	13.9 (5.6)
Matches played, mean (SD)	474 (229)	458 (250)	465 (241)
Training duration (hours per week), mean (SD)	14.6 (5.2)	14.1 (5.1)	14.3 (5.1)

*p < 0.05; **p < 0.01

^a^Self-reported constitutional malalignment (includes both varus and valgus)

^b^Pain medication includes paracetamol, NSAIDS and opioids

^c^Significant knee injury sustained during football career (time-loss of at least 3 months) 2D:4D Ratio = index–ring finger ratio

The most commonly reported KIA injections were cortisone (corticosteroid) only (*n* = 379, 70.6%), local anaesthetic injections only (*n* = 26, 4.8%) and a combination of cortisone and local anaesthetic injections (*n* = 88, 16.37%). However, the nature of 51 injections received was reported as unknown.

**Fig. 1:**
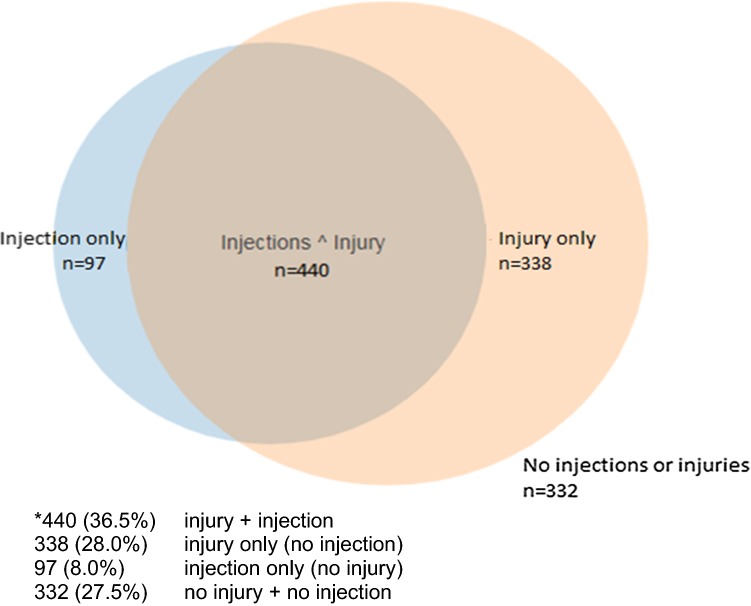
Number (%) of footballers with knee injury and/or IA injection in the all ex-footballers (*n* = 1207)*

Over half (56.6%) of the footballers who sustained a significant knee injury received an IA injection (Fig. 1). However, 22.6% of footballers who did not report a significant knee injury also received a KIA injection. Of the whole population, only 27.5% of the footballers neither sustained a significant knee injury nor received an IA injection into at least one of their knees.

Footballers had a significant increased risk of all KOA outcomes if they had received a KIA injection during their career (Table 2). These associations remained significant after adjustment for age and BMI. However, after also adjusting for knee injury, KIA injections were significantly associated with KP [OR: 1.81, 95% CI 1.40–2.34] and TKR [OR: 2.21, 95% CI 1.43–3.42], but not RKOA [OR: 1.30, 95% CI 0.85–2.01]. We ran additional analyses to look at patterns of joint space narrowing (JSN) in those who received IA injections compared to those who did not and found no association and no increase in number of compartments with JSN in those injected.

**Table 2:** KIA injection and risk of KOA outcomes in those with any injection compared with those without any injections

Outcome	Odds Ratio (95% Confidence Interval)		
	Crude	Adjusted 1^†^	Adjusted 2^‡^
Knee pain	2.76 (2.18–3.50)**	2.55 (2.01–3.25)**	1.81 (1.40–2.34)**
Radiographic KOA^#^	1.60 (1.10–2.35)*	1.66 (1.11–2.50)*	1.30 (0.85–2.01)
Total knee replacement	2.56 (1.76–3.73)**	3.23 (2.13–4.88)**	2.21 (1.43–3.42)**

^#^Radiographic KOA assessed using NLDA instrument

^†^Adjusted for: age + BMI

^‡^Adjusted for: age + BMI + Injury

*p < 0.05; **p < 0.01

As cortisone injections were the most common, we also conducted sub-group analyses where we compared cortisone-only injections (*n* = 379) to those with no injections (*n* = 663). We found similar results, and these are presented in “Appendix 1”.

We created four groups according to the number of injections, i.e., no injection (*n* = 670), 1–3 injections (*n* = 192), 4–6 injections (*n* = 103) and 7 + injections (*n* = 122). to look further at the relationship between number of IA injections and KP and TKR. We found a significant dose–response relationship between the number of IA injections and KP and TKR outcomes (*p* for trend < 0.01) (Table 3).

**Table 3:** Groups of KIA injection and risk of KOA outcomes

Outcome	Odds ratio [95% confidence interval (CI)]	
	Crude	Adjusted^†^
Knee pain		
Group 1 (no IA)	Reference	Reference
Group 2 (1–3 IA)	1.77 (1.28–2.44)	1.20 (0.85–1.70)
Group 3 (4–6 IA)	2.30 (1.50–3.55)	1.49 (0.95–2.35)
Group 4 (7 + IA)	2.83 (1.87–4.29)	1.79 (1.14–2.77)
Trend	*p* < 0.01	*p* < 0.01
Total knee replacements		
Group 1 (no IA)	Reference	Reference
Group 2 (1–3 IA)	1.03 (0.60–1.75)	1.14 (1.63–2.06)
Group 3 (4–6 IA)	2.40 (1.40–4.10)	2.01 (1.10–3.69)
Group 4 (7 + IA)	2.79 (1.72–4.53)	2.08 (1.18–3.69)
Trend	*p* < 0.01	*p* < 0.01

^#^Radiographic KOA assessed using NLDA instrument

^†^Adjusted for: age + BMI + injury

*p < 0.05; **p < 0.01

## Discussion

This is the first study to report the administration of KIA injections in professional footballers during their playing careers and to investigate their potential associations with later KOA outcomes. The main findings are: (1) 45% of ex-professional footballers reported having at least one KIA injection, predominantly corticosteroid, during their professional career; (2) the mean number of injections received over a professional football career was 7.5 (SD: 11.2) and; (3) KIA injections were administered predominantly in the context of a significant knee injury; however, even after adjusting for injury, KIA injections (predominantly cortisone injections) were associated with two subsequent KOA outcomes (KP and TKR) and showed a dose-dependent relationship but not with RKOA.

UK footballers received over twice the number of KIA injections during their playing careers than that reported in a study of 27 Brazilian ex-professional footballers and 30 non-sport controls, though that study was powered for KOA outcomes rather than IA injections, and the type of injection and injection frequency per season were unreported [12]. It was, however, similar to another study of Brazilian ex-footballers which reported 54% of players as having received IA injections. The ex-players in this sample were considerably younger (mean age 46.2 years) and played professional football in Brazil where KP management strategies including use of KIA injections may vary considerably [13]. 17% of ex-footballers in our cohort reported receiving an IA anaesthetic injection for a significant knee injury which is regarded as unsafe and associated with a high risk of major complications [5, 7, 18]. However, evidence from these previous reports is restricted to small-scale studies, some with just anecdotal reporting [5]. There is a general lack of robust information regarding the use of KIA injections such as anaesthetic and corticosteroids in the setting of knee injury and professional sports. Our results suggest a strong association, showing a dose–response relationship, between receiving KIA injections and later KP and TKR. This trend remains even after adjustment for knee injury suggesting that injections themselves may have a significant detrimental effect on the long-term knee joint health of ex-professional footballers.

Although most footballers who received an IA injection had sustained an injury, 18% received an IA injection without reporting a significant knee injury. This questions the reasoning for such IA injections and raises the possibility of players’ recall error or potentially inappropriate health professional practice in the context of professional football, for example, using IA injection for muscle strains, sprains or acute cartilage damage to minimise loss of play and performance during matches and practice sessions. It is worth addressing that if inappropriate IA injections were administered, the results presented here reflects behaviour more than 20 years ago which may differ from modern standards and practice. Nevertheless, the present data support the principle that the type and frequency of KIA injections and the indications for their use should be monitored and appropriately regulated by professional football clubs to ensure they are not administered to the detriment of the individual player’s long-term joint health.

Although IA corticosteroid injections can be beneficial to patients with KP and KOA [19–21], the potential long-term risk of cartilage degradation with repeated corticosteroid IA injections has long been a concern. This was recently highlighted in a 2-year RCT showing more cartilage attrition on certain MRI measures in people receiving regular IA injection of corticosteroid compared to placebo [9]. However, a direct causal role for IA corticosteroid injection and subsequent joint damage cannot be confirmed in our cross-sectional study design. Although we adjusted for known confounders for OA such as age, BMI and significant knee injury, there remains the possibility of confounding by indication (i.e., players with more severe knee injury and problems received more KIA injections). Also, the compartmental distribution of cartilage loss shown on X-rays was not more widespread in those who received injections, which argues against significant corticosteroid-induced cartilage changes. Our results show that the risk of KOA outcomes was greater for footballers who had received KIA injections compared to those who had not. When adjusting for knee injury, the association was significant with KP and TKR but not RKOA. This suggests that KIA injections are not an independent risk factor for RKOA, but are for KP after retirement and TKR. However, as the sample of ex-footballers who received bilateral knee radiographs was smaller (*n* = 470) compared to the entire sample (*n* = 1207), these results might be underpowered to detect radiographic structural changes. Interestingly, gout was almost twice as common in footballers who received a KIA injection. Ex-professional footballers are prone to significant knee injuries in addition to repetitive knee joint microtrauma inflicted by training and match play over a prolonged time, both of which may predispose to KOA. KOA predisposes to both urate and calcium crystal deposition, but equally urate crystal deposition in bone and cartilage predisposes to joint damage and OA. Thus, the higher prevalence of gout may reflect the amplification loop involving interplay between urate crystal formation and increasing severity of KOA [21, 22] and the presence of crystals may cause more symptoms and increase the use of intra-articular injections. Those who received KIA injections also reported using more pain medication, again suggesting more significant injury and long-term damage in those receiving injections.

There are several caveats to this work. Firstly, the study merely comments on the scale (frequency) of KIA injections used over a professional footballer’s career and presents associations between KIA injections and KOA outcomes with adjustment for a limited number of confounders. We cannot account for all confounders, particularly any significant knee injury or indeed KIA injections that may have been administered post retirement from professional football. Secondly, the study is limited by the cross-sectional study design which does not allow for any direct causal inferences to be drawn between KIA injections and KOA outcomes. Although players recalled their history of knee injections during their professional careers (predominantly in their 20s and 30s) and measured KOA in later life (mean age of cohort was 59 years) after they retired, we certainly cannot infer causality between KIA and KOA using this study design. However, our results are in line with the literature from both randomised controlled trials [9] and observational studies [23] in the general population. A long-term prospective follow-up study from playing professional football through to retirement and beyond is required to establish potential causal pathways. Thirdly, we are reliant on self-reported questionnaire (rather than previously documented) data, which is subject to recall bias. Furthermore, we were not able to distinguish between pre-, mid- and post-game injections and midweek injections as we did not include this level of granularity within the questionnaire. Lastly, ex-footballers with known health problems may have been more likely to respond to the questionnaire than ex-footballers with no known health issues, thus introducing a selection bias to these data.

## Conclusion

The study highlights the high number of KIA injections (namely cortisone injections) that were historically administered to ex-professional footballers in the UK and their association with KOA outcomes after adjustment for major confounders such as injury. However, these results are based on ex-footballer’s experience from previous decades and as such, may bear little or no relevance to current medical practices. Current WADA guidelines do not prohibit the intra-articular use of glucocorticoids; however, in the athletic context, this does not in any way suggest that their use is to be encouraged. These data provide evidence of potential sub-optimal clinical practices of using IA injections, particularly cortisone injections, to expedite players’ return to play and suggest a potential detrimental long-term impact on KOA outcomes especially KP after retirement and TKR for professional footballers. It is unclear whether these practices persist today.

## Appendix 1: KIA injection and risk of KOA outcomes in those with cortisone injections compared to those without any injections

**Table:**

Outcome	Odds ratio (95% Confidence Interval)		
	Crude	Adjusted 1^†^	Adjusted 2^‡^
Knee pain	2.53 (1.95–3.29)**	2.42 (1.86–3.17)**	2.41 (1.84–3.15)**
Radiographic KOA^#^	1.68 (1.03–2.76)*	1.60 (0.97–2.63)	1.47 (0.88–2.46)
Total knee replacement	2.26 (1.51–3.38)**	2.25 (1.47–3.36)**	2.30 (1.52–3.49)**

^#^Radiographic KOA assessed using NLDA instrument

^†^Adjusted for: Age + BMI

^‡^Adjusted for: Age + BMI + Injury

**p* < 0.05; ***p* < 0.01
